# The Impact of the Colonoscopy Starting Position and Its Potential Outcomes

**DOI:** 10.7759/cureus.25000

**Published:** 2022-05-14

**Authors:** Pallavi Shah, Nehal Patel, Alhareth Alsayed, Steven Miller, Nitish Singh Nandu

**Affiliations:** 1 Internal Medicine, Rosalind Franklin University, North Chicago, USA; 2 Gastroenterology, Captain James A. Lovell Federal Health Care Center, North Chicago, USA; 3 Internal Medicine, Chicago Medical School, Chicago, USA; 4 Medical Oncology, CGH Dixon Medical Center, Dixon, USA; 5 Biostatistics and Epidemiology, Rosalind Franklin University, Chicago Medical School, North Chicago, USA; 6 Internal Medicine, Chicago Medical School, Rosalind Franklin University, North Chicago, USA

**Keywords:** left lateral colonoscopy, colon cancer surveillance, prone, colonoscopy complications, prone colonoscopy

## Abstract

Based on the literature review, many studies have been inconclusive in regards to adenoma detection and procedural positioning during a colonoscopy. Scope looping can make cecal intubation challenging, changing the positioning of the patient and application of external abdominal pressure can overcome this difficulty. A colonoscopy in a prone position can overcome these challenges and reduce cecal intubation time. It can thus improve the safety of the patient and the staff by minimizing the movement of a sedated patient.

## Introduction

Based on the literature review, studies have shown mixed results regarding adenoma detection and procedural positioning during a colonoscopy [1-7]. Scope looping can make cecal intubation challenging, repositioning the patient and application of external abdominal pressure are maneuvers used to overcome this difficulty. A colonoscopy in the prone position may overcome these challenges and reduce cecal intubation time. Thus, it can be safer for the patient and the procedural staff by minimizing the movement of a sedated patient.

We hypothesized that performing a colonoscopy entirely in a prone position would utilize the patient’s weight to help maintain external pressure. This would allow the scope to remain straight thereby preventing looping and reducing the need for external abdominal pressure and patient repositioning. Based on a smaller sample of observation by the endoscopist, we additionally hypothesized that the adenoma detection (AD) and polyp detection (PD) might be higher when a colonoscopy was performed in the prone position.

## Materials and methods

The IRB approval was given by the Edward Hines Veterans Administration Hospital Institutional Review Board (IRB) (PROJECT ID: 17-053).

Objective

Evaluate the impact of the prone or left lateral starting position of colonoscopy on the number of adenomas detected, and the cecal intubation time. Secondary outcomes measured included the need for external abdominal pressure and repositioning of a sedated patient to achieve cecal intubation.

Design

A prospective single-blinded, single-operator, randomized controlled trial (RCT) was conducted and 291 consenting patients presenting for a colonoscopy to the gastroenterology department were enrolled. The study was conducted at the Captain James A. Lovell Federal Health Care Center, patients were: retirees, veterans, active-duty military personnel, and their dependents. The following inclusion and exclusion criteria were used.

Inclusion criteria: Patients >18 years and <90 years of age, presenting to the Captain James A. Lovell Federal Health Care Center, Gastroenterology suite for all colonoscopy indications under all types of sedation protocols.

Exclusion criteria: Pregnant women, military recruits, those who can’t participate in their assigned position and those unable or unwilling to sign informed consent, cognitively impaired, and procedures that were aborted due to very poor bowel prep.

The selected patients were randomly assigned to a starting colonoscopy position (left lateral or prone), with the application of external abdominal pressure, and repositioning was needed to achieve successful cecal intubation.

Data collection

Information on the age, number of comorbidities, gender, obstructive sleep apnea, history of abdominal or pelvic surgeries, and procedural indication were obtained from the patients during the interview with the physician prior to the procedure. Information on the type of scope used, duration and type of sedation, medications used, cecal intubation time, quality of prep, need for repositioning or external abdominal pressure, and number and type of polyps, and withdrawal time was obtained during the procedure by the collaboration of the physician, nursing, and anesthesiologist.

Statistical analysis

Categorical variables were tested for association using Pearson’s chi-squared test. Continuous variables were analyzed using student’s t-test.

## Results

The study’s primary endpoints were AD, PD, and cecal intubation time. Two hundred ninety-one patients were recruited for the study. In total four patients were excluded. Two patients were excluded due to poor colonoscopy preparation, one patient was excluded due to obstructing mass preventing cecal intubation, and one patient was excluded who declined the assigned position after enrollment. Thus, leaving 287 patients included in the study.

A total of 134 male and 13 female subjects were assigned to the left lateral position. A total of 117 males and 23 females were assigned to the prone position. There were no significant differences between the two groups in terms of age, sex, BMI, sleep apnea, indications for colonoscopy, history of abdominal/pelvic surgery, the number of comorbid conditions, duration of sedation, and withdrawal time as summarized in Table 1.

**Table 1:** Comparison of the patient demographic data distribution between the prone and the lateral groups BMI: body mass index; OSA: obstructive sleep apnea

Patient Demographics								
	Lateral		Prone					
	Total		Total		Test statistic	df	p-value	Effect size (v)
Gender	134 Male		117 Male		Chi Square = 3.761	1	0.052	-0.114
	13 Female		23 Female					
OSA	47		41		Chi Square = 0.393	1	0.531	-0.037
History of abdominal/pelvic surgery	38		37		Chi Square = 0.012	1	0.911	0.007
	Mean	Std. deviation	Mean	Std. deviation	Test statistic	df	p-value	Effect size (d)
Age	59.86	13.188	59.19	12.572	t-test = 0.446	285	0.656	0.53
Number of Comorbidities	1.37	1.278	1.17	1.217	t-test = 1.375	285	0.17	0.162
BMI	29.7631	5.58356	29.6737	4.96703	t-test = 0.143	285	0.886	0.017

Primary outcomes

AD was categorized into two groups in order to identify an association with the starting colonoscopy position. The first category was 0-2 polyps detected (recommended surveillance interval of 7 years) and the second group was three or more polyps detected (recommended surveillance interval of 3-5 years). There was no statistically significant association between AD category and starting colonoscopy position, as seen in Table 2.

**Table 2:** Comparison of the total number of adenomas detected, number of high-grade polyps, and number of proximal hyperplastic polyps among the lateral and the prone groups

Polyps detected						
	Lateral	Prone	Test statistic	p-value	df	Effect size (phi)
Total number of adenomas			Chi Square = 2.605	0.107	1	-0.095
0-2	114	119				
3 or more	33	21				
Number of high-grade polyps			Chi Square = 2.578	0.108	1	-0.095
Not detected	101	108				
Detected	46	32				
Number of proximal hyperplasic polyps			Chi Square = 2.539	0.111	1	0.094
No polyp	138	124				
More than 1 polyp	9	16				

Secondary outcomes

There was no statistically significant difference in the procedural indications for colonoscopy (p=0.947), medical history of obstructive sleep apnea (p=0.531), and history of abdominal or pelvic surgery (p=0.911) (Table 1).

The detection of high-grade polyps (p=0.108) and proximal hyperplastic polyps (p=0.094) in either position was similar. However, it was noted that the high-grade lesions were more likely to be picked up in the lateral position as compared to the prone position (Table 2). The need to apply external abdominal pressure to achieve cecal intubation was higher in the left lateral position as compared to the prone position (109 left lateral versus 44 prone (p <0.001)). These results are summarized in Table 3.

**Table 3:** Comparison of peri-procedural application of external abdominal pressure and the need to reposition among the lateral and the prone groups

Peri-procedural demographics						
	Lateral	Prone		P-value	df	Effect size (v)
Need for external abdominal pressure	108	44	Person Chi Square	p<0.001	1	52.989
Need to re-position	43	16	Person Chi Square	p<0.001	1	14.594

There was no statistically significant difference in the type of scope (adult vs. pediatric colonoscope) used in either position (Table 4).

**Table 4:** Comparison of the pediatric and adult colonoscope use among the lateral and the prone groups

Scope demographics						
	Lateral	Prone	df	p-value	Effect size (phi)	Test statistic
Type of scope			1	0.237	0.07	Chi-squared 1.396
Pediatric	93	79				
Adult	54	61				

The mean duration of sedation was 31 minutes in the lateral position compared to 28 minutes in the prone position (p=0.052). The mean cecal intubation time was 7 minutes in the lateral position and 6.5 minutes in the prone position (p=0.247). The mean withdrawal time was 19 minutes in the lateral position and 11 minutes in the prone position (p=0.065) (Table 5).

**Table 5:** Comparison of duration of sedation, cecal intubation time, and withdrawal time (in minutes) among the lateral and the prone groups

Peri-procedural demographics								
	Lateral		Prone					
	Mean	Std	Mean	Std	Test statistic	df	p-value	Effect size (d)
Duration of sedation (mins)	31.37	12.575	28.49	12.385	t-test = 1.955	285	0.052	0.231
Cecal intubation time (mins)	7.24	5.755	6.49	5.308	t-test = 1.16	285	0.247	0.137
Withdrawal time (mins)	19.61	11.446	17.14	11.086	t-test = 1.855	285	0.065	0.219

There was no statistical difference in the quality of colon preparation in either position (p=0.895). There was no statistical difference in the type of sedation used and the route of administration in either group (Tables 6-7).

**Table 6:** Comparison of quality of the colonoscopy preparation and the type of sedation used among the lateral and the prone groups

Peri-procedural demographics						
	Lateral	Prone	Test statistic	df	p-value	Effect size (v)
Quality of colon prep			Chi Square = 0.221	2	0.895	0.028
Good 2	108	101				
Adequate 1	35	36				
Poor 0	4	3				
Sedation type			Chi Square = 0.184	1	0.668	-0.025 (phi)
Mac	16	13				
IV	131	126				

**Table 7:** Comparison of peri-procedural analgesic use among the lateral and the prone groups

Peri-procedural analgesic demographics								
	Lateral		Prone					
	Mean	Std deviation	Mean	Std deviation	Test statistic	df	p-value	Effect size (d)
Meperidine (mg)	45.93	20.875	46.61	19.696	t-value = -0.285	285	0.776	-0.034
Diphenhydramine (mg)	11.39	25.547	7.64	17.685	t-value= 1.452	260.588	0.148	0.17
Midazolam (mg)	3.97	1.912	4.09	1.885	t-value = -0.535	285	0.593	-0.063
Propofol (mg)	25.86	88.377	22.27	81.005	t-value = 0.358	285	0.721	0.42

## Discussion

Colorectal cancer (CRC) is the third most common cancer in the United States. It is estimated that nearly 149,500 new CRC cases will be diagnosed this year, accounting for 7.9% of all new cancer cases. Moreover, there will be 52,980 deaths this year from CRC which accounts for 8.7% of all cancer deaths [8].

A colonoscopy is considered the gold standard for colon cancer screening. Adenoma detection rate (ADR) has been established as a key performance indicator for the quality of colonoscopy [9]. Higher ADRs in screening colonoscopy were associated with lower lifetime risks of CRC and its mortality [10-12]. Published data shows a higher rate of interval CRC with a lower ADR [12]. A careful examination of the colonic flexures and folds, suctioning, and cleaning of residue and debris, along with a high-quality withdrawal technique is associated with a higher ADR [3,13-15].

Based on an observation of a smaller sample of patients, we hypothesized that performing a colonoscopy in a prone position opens the colonic folds allowing better visualization of the colon, thus improving AD and allowing for cecal intubation without the use of external abdominal pressure or patient repositioning. Per the literature review, there were no studies that assessed the impact of a starting colonoscopy position on AD. Our study found AD to be similar in both prone and lateral positions. A study by Ou et al. reported no effect on ADR and polyp detection rate (PDR) with position changes during colonoscopy withdrawal when the baseline ADR is above the recommended standard [3]. A systematic review by Zhao et al. involving seven RCTs reported improvement in ADR, and PDR in four of the RCTs, while three parallel-group RCTs did not confirm its effectiveness [7]. A meta-analysis by Li et al., which collected data from five different studies reported an increase in the ADR and no increase in PDR with dynamic position changes [6]. Thus, studies have shown mixed results regarding the effect of position changes during a colonoscopy on the PDR and ADR over the usual practice [1,3,5,7].

In our study, cecal intubation time was similar in both prone and left lateral starting positions. Vergis et al. reported no benefit from a prone starting position over a conventional left-sided starting position, additionally, the prone starting position led to an increase in time to reach the cecum [5]. A study by Uddin et al. found that the colonoscopy in a prone position result in significantly shorter cecal intubation time and decreased need for repositioning in patients with BMI > 30 kg/m^2^ [16]. Vergis et al. compared the impact of starting in right versus left lateral starting position and found that the right lateral starting position was more comfortable for the patient and had a quicker cecal intubation time [17]. Achieving cecal intubation is challenging in patients with a redundant colon and those with a BMI > 30 kg/m^2^ because it increases the likelihood of scope looping which prevents cecal intubation. It is speculated that the prone position redistributes abdominal pressure and prevents looping of the scope [16]. In our study, colonoscopies in a prone position achieved cecal intubation with minimal need of repositioning a patient and application of external abdominal pressure when compared to the left lateral position.

The task of repositioning a sedated patient and applying external abdominal pressure can place both the patient and the medical staff at risk for musculoskeletal injuries [18]. Many studies have demonstrated injuries associated with repositioning of the patient. Repositioning requires a patient to be grasped under the axilla and maneuvered, this can compress the underlying brachial plexus and arteries which can cause injury [19]. Repositioning a sedated patient is associated with an inherent risk to the patient such as: falling out of the procedural bed, dislodgement of an endotracheal tube, loss of IV access, vascular compression, as well as muscle, nerve, and skeletal injuries.

There is also a plethora of research indicating that patient repositioning exposes the endoscopy team to a higher risk of occupational injury [19-25]. Movements such as lifting, bending, and repetitive movements with awkward positions are regularly occurring when caring for patients [24]. The US Bureau of Labor Statistics noted that healthcare workers rank above other strenuous professions such as truck drivers, laborers, and janitors for incidence of occupational injuries. Musculoskeletal disorders, especially back injuries that are associated with patient handling tasks, contribute to this injury prevalence [26]. A systematic review by Schlossmacher and Amaral (2012) demonstrated that 9.1% of lower back pain reported by nurses was secondary to patient repositioning [27]. Murty reported that the work demands placed on endoscopy nurses were strenuous; the neck, back, and shoulders were exposed to a higher risk of musculoskeletal injury due to the physical demands of the job [20]. Musculoskeletal injuries cause a significant burden to the paramedical staff and can impact the quality of life and time spent away from work.

Limitations

As the study predominately included males, the impact of this positioning on the female gender was not clearly evaluated. Furthermore, the application across different providers with varying levels of AD rate is unclear and further studies are needed to evaluate the effect of starting position of colonoscopy in providers with different AD rates.

## Conclusions

From this study, we noted that the need for external abdominal pressure or the need to reposition is significantly lower in the prone starting position as compared to the left lateral starting position; thus, minimizing the chance of musculoskeletal-related injuries making it safer for patients and the endoscopy staff.

A conditional conclusion that can be drawn from this study is if the endoscopist has an adequate ADR, which was 43 in this study, the starting position of colonoscopy will not affect the adenoma detection. However, this requires future investigation where this hypothesis is tested in providers with different levels of ADR.
